# A Tale From the Early Stone Age: Pediatric Ureterolithiasis as Appendicitis Mimic - A Case Report and Management Overview

**DOI:** 10.7759/cureus.10637

**Published:** 2020-09-24

**Authors:** Neil P Larson, Rachel E Bridwell, Michael J Yoo

**Affiliations:** 1 Emergency Medicine, Brooke Army Medical Center, Fort Sam Houston, USA

**Keywords:** ureterolithiasis, pediatric urinary stone disease

## Abstract

Ureterolithiasis in the pediatric population is uncommon and may mimic acute abdomen. While the majority of small stones pass spontaneously, complications may ultimately warrant surgical intervention. As the majority of affected patients have a predisposing condition, targeted therapy with close and consistent follow-up may prevent recurrence, emphasizing timely diagnosis. The authors present the case of a previously healthy 13-year-old boy with eight days of right lower quadrant abdominal pain and emesis, who was found to have a distal ureteral stone necessitating surgical intervention.

## Introduction

Unlike in the adult population, pediatric urinary stone disease is an uncommon emergency department (ED) diagnosis. Patients less than 18 years of age comprise only 2%-3% of the urinary stone patient population, with the estimated incidence of pediatric urinary stone disease at approximately 59.5 cases per 100,000 person years [1,2]. Incidence increases with age peaking in adolescence, and there is a slight female predominance [2,3]. Caucasians consist of the vast majority (88%) of pediatric urinary stone patients [2,4]. Flank or abdominal pain is the most common presenting symptom followed by gross hematuria [4,5].

## Case presentation

**Figure 1:**
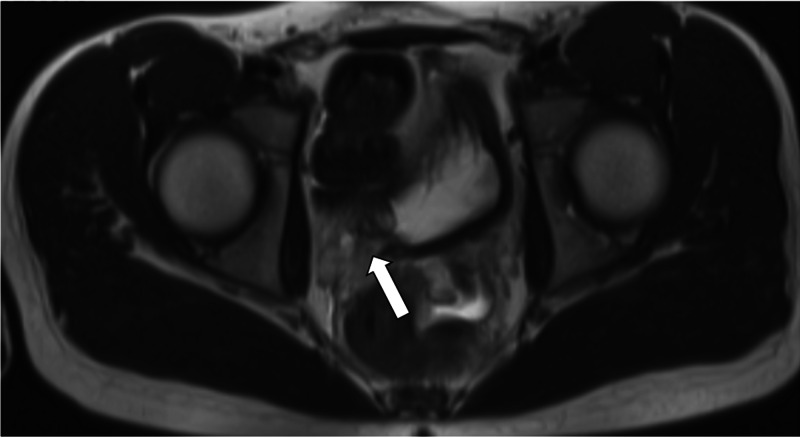
Axial view of HASTE pelvic MRI demonstrating approximately three millimeter ureteral stone near the right ureterovesicular junction with mild ureteral dilatation (white arrow). HASTE, Half-Fourier acquisition single-shot turbo spin echo

A 13-year-old previously healthy African American male presented to the ED for eight-day duration of right lower quadrant pain and new onset intractable nausea and vomiting. He was previously evaluated at another facility four days prior and discharged home with symptomatic therapy. On review of systems, the patient denied recent travel, fever, abdominal trauma, previous abdominal surgeries, blood dyscrasias, or diarrhea. The patient’s initial vital signs were blood pressure of 93/61 mm Hg, heart rate of 123 beats per minute, respiratory rate of 20 breaths per minute, and oxygen saturation of 98% in room air with a temperature of 98.7 degrees Fahrenheit. Physical exam was notable for mild right lower quadrant tenderness with rebound, but negative for costovertebral angle tenderness or testicular tenderness. Laboratory analysis reviewed a white blood cell count of 15,200 cells/µL with an 85% neutrophilic predominance, though comprehensive metabolic panel and lipase were unremarkable. Specifically, the patient's blood urea nitrogen (BUN) and creatinine were 17.9 mg/dL and 0.93 mg/dL, respectively, and the urinalysis was without blood, nitrite, or leukocyte esterase, but did contain 1+ ketones and a mildly elevated specific gravity of 1.031 (normal range 1.005-1.025). The patient underwent evaluation for acute appendicitis, and while the abdominal ultrasound (US) was non-diagnostic, MRI demonstrated a normal appendix, right perinephric fat stranding, mild hydronephrosis, and a 3 millimeter (mm) ureterolith at the right ureterovesicular junction (Figure 1). The patient received 1,000 mL of Ringer’s lactate solution, 650 mg of intravenous acetaminophen, and 4 mg of intravenous ondansetron, with resolution of tachycardia and improvement of symptoms. Urology was consulted for further management, and the patient was admitted to the hospital for trial of medical therapy including medical expulsive therapy. However, due to refractory pain and emesis without stone passage, coupled with possible ureteral obstruction, the patient subsequently underwent successful cystoscopy, ureteroscopy, laser lithotripsy, and ureteral stent placement. The patient was discharged after an uneventful postoperative course, and noted resolution of symptoms at postoperative follow-up appointment.

## Discussion

The differential diagnosis of right lower quadrant abdominal pain in the pediatric population is broad and includes appendicitis, testicular or ovarian torsion, infection, and referred musculoskeletal pain from the lower extremities. Although uncommon, ureterolithiasis may present similar to an acute surgical abdomen, with patients exhibiting acute onset of nausea and vomiting, intractable pain, and peritoneal signs. Workup should begin with a thorough physical exam, including evaluation of the back, abdomen, hip, and pelvis, potential hernia sites, and genitals as necessary. While non-specific, new onset gross or microscopic hematuria on urinalysis supports the diagnosis of a kidney stone [6]. Additionally, a urinalysis and urine culture should be obtained to rule out a concomitant infection. A renal function panel is essential to evaluate overall renal performance. Ultimately, imaging is needed to confirm a suspected diagnosis of acute urinary stone. Although commonly ordered due to high sensitivity and specificity (up to 98% and 99%, respectively), CT of the abdomen and pelvis should be avoided, if possible, in pediatric patients due to radiation exposure and significantly higher study cost compared to US. As pediatric body habitus is generally more amenable to US, with a reported sensitivity and specificity of 76% and 100%, renal US remains the initial preferred imaging modality in evaluating for pediatric urinary stones. However, as with our patient, smaller stones and distal ureteral stones are more likely to be missed on US [7]. With an estimated sensitivity and specificity of 82% and 98%, MRI can also be used as a radiation sparing modality [8]. However, due to duration of study, which may potentially require sedation, and significantly increased cost in comparison to both US and CT, MRI only accounts for approximately 2% of imaging in pediatric urinary stone patients, and is more difficult to obtain in the ED [8,9]. As such, initiating imaging workup for suspected acute urinary stone with renal US in the pediatric population is the recommendation of the authors as well.

The initial treatment of pediatric acute urinary stone disease should include aggressive hydration, antiemetics for nausea, and analgesia. According to recent American Urological Association (AUA) guidelines, ureter stone sizes of less than or equal to 10 mm in pediatric patients may be observed and trialed with medical therapy [10]. While other studies and hospital guidelines may vary according to recommended management of stones based on size alone, a recent study in accordance with AUA guidelines suggested that stones less than 6.7 mm in size have greater than an 80% chance of spontaneous passage [11]. Subsequently, a hemodynamically stable patient with a small, uncomplicated stone with well-controlled pain and nausea can be discharged with close urology follow-up. Barring contraindications, outpatient symptomatic therapy includes non-steroidal anti-inflammatory drugs (NSAIDs), acetaminophen, opioid analgesia for breakthrough pain, medical expulsive therapy, and a urine strainer for stone capture. However, underlying renal disease, solitary kidney state, larger stone size, complete ureter obstruction, concomitant urinary tract infection, and struvite stones necessitate urological evaluation for surgical intervention due to an increased risk for inability to pass stone, acute decompensation, and long-term morbidity [10,12-15]. Intractable pain and failure of stone passage with trial of medical therapy, as experienced by our patient, similarly merit urology evaluation [4,10]. Similar to the adult population, calcium oxalate stones are the most common, followed by calcium phosphate stones trailed by smaller portions of several other types [16]. Although any pediatric patient can develop stone disease, the majority of pediatric stone patients have underlying predisposing conditions, including metabolic abnormalities (hypercalciuria, hyperoxaluria, among several others), genitourinary tract structural abnormalities, and urinary tract infections [3,5,16]. Unfortunately, as the majority of pediatric patients experience recurrent episodes of stones, timely diagnosis and tailored treatment targeting the underlying predisposition should be aggressively pursued in conjunction with urology.

## Conclusions

Pediatric urinary stone disease is an uncommon diagnosis and may mimic more common causes of acute abdomen to include appendicitis. In addition to a thorough physical examination, laboratory workup including urinalysis and imaging to include renal US when possible will aid in diagnosis. While hydration, analgesia, and antiemetics are the mainstay of treatments and most stones pass spontaneously, emergent urology consultation for procedural intervention is warranted in multiple circumstances to prevent complications. Predisposing conditions including metabolic abnormalities are present in the majority of patients and warrant follow-up and therapy targeted at stone recurrence prevention when possible.
